# Synergistic Effects of Hyperandrogenemia and Obesogenic Western-style Diet on Transcription and DNA Methylation in Visceral Adipose Tissue of Nonhuman Primates

**DOI:** 10.1038/s41598-019-55291-8

**Published:** 2019-12-17

**Authors:** Lucia Carbone, Brett A. Davis, Suzanne S. Fei, Ashley White, Kimberly A. Nevonen, Diana Takahashi, Amanda Vinson, Cadence True, Charles T. Roberts, Oleg Varlamov

**Affiliations:** 1 Department of Medicine, Knight Cardiovascular Institute, Oregon Health & Science University, Portland, OR USA; 2 Department of Molecular and Medical Genetics, Oregon Health & Science University, Portland, OR USA; 3 Department of Medical Informatics and Clinical Epidemiology, Oregon Health & Science University, Portland, OR USA; 4 Division of Genetics, Oregon National Primate Research Center, Beaverton, OR USA; 5 Division of Cardiometabolic Health, Oregon National Primate Research Center, Beaverton, OR USA; 6 Division of Reproductive and Developmental Sciences, Oregon National Primate Research Center, Beaverton, OR USA

**Keywords:** Metabolic syndrome, DNA

## Abstract

Polycystic ovary syndrome (PCOS) is a major reproductive disorder that is responsible for 80% of anovulatory infertility and that is associated with hyperandrogenemia, increased risk of obesity, and white adipose tissue (WAT) dysfunction. We have previously demonstrated that the combination of chronic testosterone (T) treatment and an obesogenic Western-style diet (WSD) exerts synergistic functional effects on WAT, leading to increased lipid accumulation in visceral adipocytes by an unknown mechanism. In this study, we examined the whole-genome transcriptional response in visceral WAT to T and WSD, alone and in combination. We observed a synergistic effect of T and WSD on gene expression, resulting in upregulation of lipid storage genes concomitant with adipocyte hypertrophy. Because DNA methylation is known to be associated with body fat distribution and the etiology of PCOS, we conducted whole-genome DNA methylation analysis of visceral WAT. While only a fraction of differentially expressed genes also exhibited differential DNA methylation, *in silico* analysis showed that differentially methylated regions were enriched in transcription factor binding motifs, suggesting a potential gene regulatory role for these regions. In summary, this study demonstrates that hyperandrogenemia alone does not induce global transcriptional and epigenetic response in young female macaques unless combined with an obesogenic diet.

## Introduction

Polycystic ovary syndrome (PCOS) is a major reproductive disorder affecting 5–20% of women, depending on the diagnostic criteria employed^1^. The principal symptoms of PCOS include hyperandrogenemia and infertility. Additionally, women with PCOS exhibit increased rates of obesity and insulin resistance and a higher risk of developing gestational and subsequent type-2 diabetes^2–5^. However, the role of obesity in PCOS remains unclear. While roughly 50% of PCOS patients are obese, obesity does not appear to be required for the PCOS phenotype, and there is some evidence that the increased rate of obesity in PCOS reflects referral bias^6–8^. On the other hand, weight loss and insulin-sensitizing drugs are some of the most successful treatments for PCOS-associated infertility^9^, arguing for a more causative role of metabolic dysfunction in PCOS pathology. Studies in humans and rodent models of PCOS indicate that hyperandrogenemia is associated with the development of white adipose tissue (WAT) dysfunction, which includes increased visceral adiposity^10–12^ and visceral^13–17^ and subcutaneous^10,18,19^ adipocyte hypertrophy. Furthermore, both hyperandrogenemia and obesity have been found to independently reduce fertility through alterations in the hypothalamic-pituitary-ovarian axis^20–22^. However, whether obesity and hyperandrogenemia exert additive or synergistic effects on metabolic and reproductive functions remain unclear and challenging to study in the human population.

Animal studies have shown that prenatal programing by maternal androgens predisposes offspring to increased adiposity and other metabolic features of PCOS, as demonstrated in nonhuman primate (NHP)^23,24^, sheep^25,26^, and rodent^27,28^ models of PCOS. Consistent with animal models, clinical studies have demonstrated that offspring of women with PCOS develop increased body weight, reflecting the significance of the intrauterine environment and genetic factors in the etiology of PCOS^29–31^. Furthermore, it has been demonstrated that peripubertal obesity is associated with hyperandrogenemia and hyperinsulinemia in young girls^32–34^, indicating that adolescence, in the presence of obesogenic factors, is a particularly vulnerable stage for the development of PCOS. However, there is a paucity of animal studies that address the mechanisms of early-life androgen exposure on female metabolism and reproduction.

To better understand the relationship between peripubertal hyperandrogenemia and obesity, we developed a NHP model in which we could test the effects of mild hyperandrogenemia and diet on systemic metabolic and WAT-specific parameters. Prepubertal female rhesus macaques were exposed to either hyperandrogenemia (*T*), a high-fat, calorie-dense (per mass of diet) Western-style diet (*WSD*), or a *T* + *WSD* combination for 3 years^35,36^. *T* + *WSD*-treated animals were more insulin-resistant, and gained more fat mass over the first 3 years of treatment compared to the control (*C*), *T*, and *WSD* groups (Supplementary Table S1). Furthermore, the *T* + *WSD* combination induced greater alterations in WAT function compared to the other groups, including an increase in visceral adipocyte size, reduced basal lipolysis in subcutaneous and visceral (omental) WAT, and increased insulin-stimulated free fatty acid (FFA) uptake in omental WAT^36^. All experimental animals in this study experienced ovulatory menstrual cycles to some extent; however, mild hyperandrogenemia alone and in combination with a WSD impaired several markers of normal ovarian and uterine function^37,38^, which ultimately resulted in increased time to pregnancy (*T* and *T* + *WSD* groups), reduced pregnancy rates (*WSD* and *T* + *WSD* groups), and increased early pregnancy loss^39^. Collectively, these studies suggest a synergistic effect of *T* and *WSD* treatment on many metabolic and reproductive outcomes.

While most human studies to date have focused on subcutaneous WAT because of its relative ease of access^40–43^, women with PCOS display increased intra-abdominal visceral adiposity that is linked to the pathophysiology of insulin resistance and metabolic syndrome^10–12^. However, the mechanisms driving excess visceral adiposity in women with PCOS and in relevant animal models remain largely unknown. Previous studies suggest that DNA methylation is involved in the regulation of WAT transcriptional profiles, contributing to body fat distribution^44^ and modulating the pathophysiological response to obesity^45^. For example, studies of obese women two years after weight loss gastric bypass surgery revealed differential methylation of adipogenic genes, which may contribute to resistance to weight loss^46^. Furthermore, altered DNA methylation in genes involved in inflammation and glucose and lipid metabolism has been reported in peripheral and umbilical cord blood, as well as in various tissues of women with PCOS^47^. However, relatively few studies to date have addressed DNA methylation changes in WAT of women with PCOS^40,42,43^. For example, a recent genome-wide study using subcutaneous WAT of women with PCOS identified a set of genes that displayed both altered DNA methylation and mRNA levels. These genes were associated with pathways involved in inflammation, metabolism and adipogenesis^42^. However, no studies to date addressed a relationship between DNA methylation, gene expression, and adipocyte function in women with PCOS or in animal models.

To explore the potential mechanisms contributing to the synergistic effects of *T* and *WSD* treatment, we examined transcriptional and epigenetic changes in visceral WAT. In this study, we demonstrate that, similar to the effects of *T* and *WSD* on systemic metabolic and WAT-specific parameters described in our previous studies^35,36^, the combination of hyperandrogenemia and WSD induced synergistic effects on both gene expression and DNA methylation in visceral WAT of rhesus macaques. We additionally describe the relationship between differential transcription, DNA methylation, and adipocyte size, and provide *in silico* analysis, suggesting that *T* + *WSD*–induced differential methylation may affect transcription factor (TF) binding sites that are computationally predicted to act as the *trans*-regulatory elements.

## Results

### The combination of hyperandrogenemia and WSD elicits a greater transcriptional response than either treatment alone

**Figure 1:**
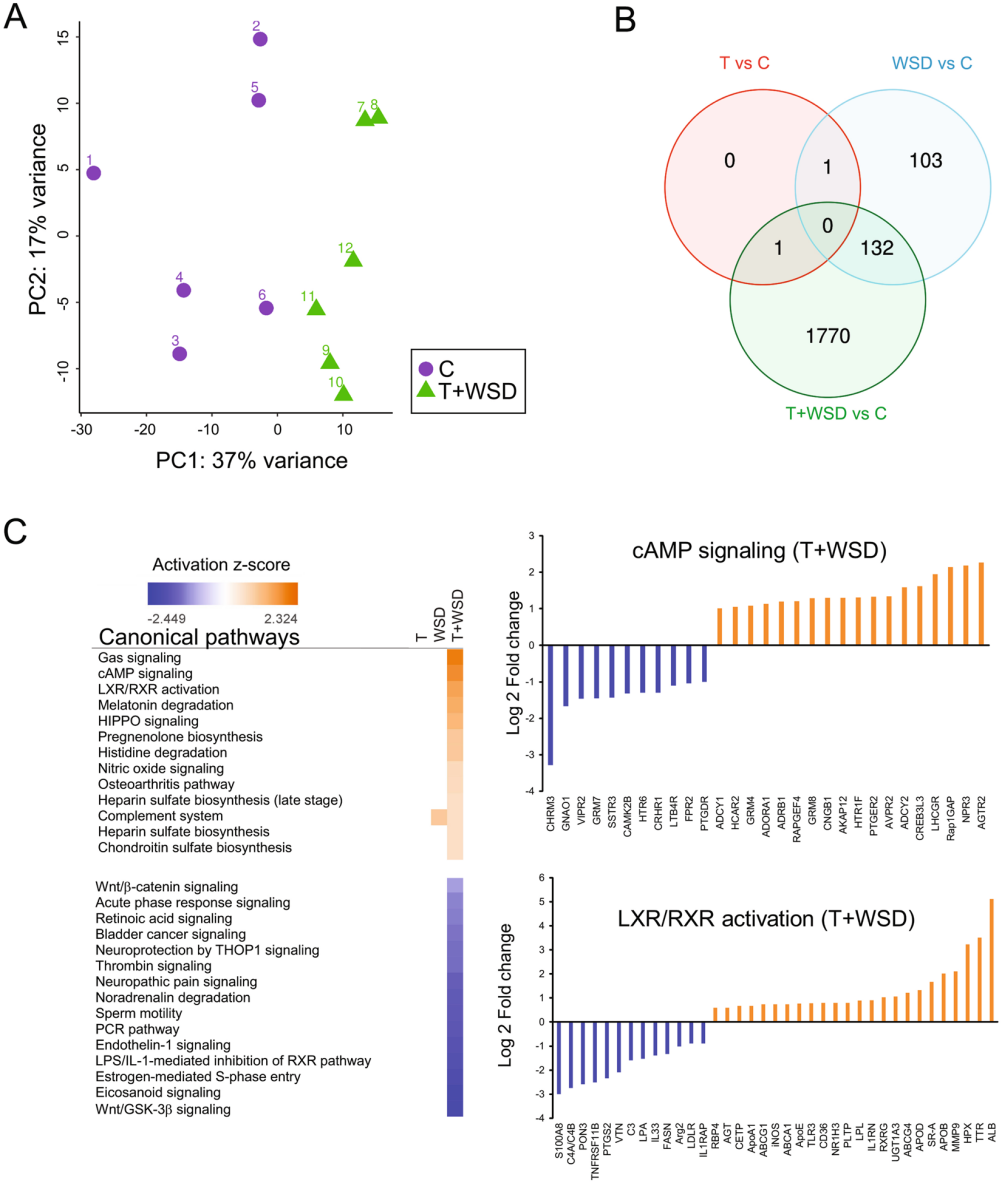
Individual and combined effects of *T* and *WSD* on gene expression in omental WAT. (**A**) Principal component analysis (PCA) of gene expression including the 500 most variable genes among the 12 samples (n = 6 samples per each group) showing segregation of controls (*C*) and *T* + *WSD*. (**B**) Venn diagram showing the overlap between DEGs in the three independent comparisons: *T* (red), *WSD* (blue) and *T* + *WSD* (green) using a fold change cutoff of 1.5 and an FDR cutoff of 0.05. (**C**) Pathway analysis using IPA of DEGs in the *T* + *WSD* group. IPA assigns an activation score based on biological relevance and the number of genes in the canonical pathway. “Log Ratio” is calculated as Log_2_ (fold change) in gene expression compared to control. Orange, activated pathways; blue, inhibited pathways; no color, no change in activation state.

To address the potential molecular mechanisms underlying WAT dysfunction in the presence of hyperandrogenemia and/or WSD, visceral WAT biopsies were collected after a 3-year treatment period from 6 animals from each of the four experimental groups (controls (*C*), *T, WSD*, and *T* + *WSD*, see methods and Supplementary Table S1) and subjected to RNAseq analysis. Principal component analysis (PCA) showed that global patterns of gene expression were significantly different between the combined treatment (*T* + *WSD*) and control groups, with the *C* and *T* + *WSD* groups clearly segregating into distinct areas (Fig. 1A). The other two groups showed less separation from the *C* group, although they still appeared as separate groups (Supplementary Fig. S1A). To determine the relative contributions of hyperandrogenemia and WSD, we independently compared the three intervention groups to controls and identified differentially expressed genes (DEGs; FDR < 0.05; −1.5 > Fold Change > 1.5). We refer to these comparisons as *T*, *WSD*, *T* + *WSD* hereafter. As expected, we identified a substantially larger number of DEGs in the *T* + *WSD* group (n = 1,903) than in the *WSD* (n = 236) and *T* (n = 2) groups (Fig. 1B; Supplementary Tables S2–S4). These findings indicate a synergistic effect of *T* and *WSD* on the WAT transcriptome, as most differential gene expression was observed in the combination treatment. One possible limitation of our study is the relatively small sample size that might limit our power to detect smaller effects caused by WSD and T alone. Nevertheless, we did identify biologically relevant DEGs whose regulation was driven exclusively by WSD (i.e., shared between *WSD* and *T* + *WSD*), including *GPT* (glutamic-pyruvic transaminase) and *MOGAT1* (monoacylglycerol O-acyltransferase 1) (Supplementary Tables S2 and S3). Altered expression of these genes has been reported in subcutaneous WAT from PCOS women^42^. Furthermore, *HCAR*2 (hydroxycarboxylic acid receptor 2) was significantly upregulated in both *T* and *T* + *WSD* groups, suggesting a WSD-independent effect of hyperandrogenemia (Supplementary Tables S2 and S4). Ingenuity pathway analysis (IPA) of DEGs in the *T* + *WSD* group identified activation of the G-coupled receptor G_as_/cAMP, LXR/RXR, and HIPPO signaling pathways, whereas the eicosanoid, Wnt signaling, and retinol biosynthesis pathways were downregulated (Fig. 1C and Supplementary Table S5). No significantly differentially regulated pathways were identified in the *WSD* and *T* groups.

### Combined *T* + *WSD* has a synergistic effect on a subset of genes

In order to identify genes for which the combined *T* + *WSD* treatment had a synergistic effect on expression, we looked at the interaction term of testosterone and WSD, utilizing the DESeq. 2 package^48^. Genes exceeding a significance threshold of an adjusted p-value < 0.1 in the interaction and also showing the same direction of log fold change in expression in the “*T* vs *C*” and “*T* + *WSD* vs *WSD*” comparisons were considered to be significantly synergistically regulated. We identified 8 up-regulated genes (*CDC42BPA*, *ERC1*, *FTL*, *KLF8, NIN*, *RYR2*, *VPS13C*, and *ZNF589*) and 3 down-regulated genes (*CCT8*, *DDT* and *PHB2*) as a result of *T* + *WSD* treatment (Table 1, Supplementary Table S6 and Supplementary Fig. S2A). We additionally performed pathway analysis including genes possibly showing a synergistic effect due to treatment, for which the unadjusted p-value was <0.1, in order to identify possible trends. Interestingly, this analysis showed that genes synergistically downregulated were enriched in cholesterol biosynthesis, glycolysis and gluconeogenesis, and SREBP signaling pathways (Supplementary Fig. S2B).

**Table 1:** Synergistic regulation of gene expression by T and WSD in omental WAT.

Gene name	Gene description	Gene ID	Log2FC	p-value
**Upregulated genes**				
RYR2	Ryanodine Receptor 2	ENSMMUG00000001060	2.22	0.0328
FTL	Ferritin Light Chain	ENSMMUG00000003909	0.77	0.0246
KLF8	Kruppel Like Factor 8	ENSMMUG00000014678	0.65	0.0925
ZNF589	Zinc Finger Protein 589	ENSMMUG00000021607	0.61	0.0796
NIN	Ninein	ENSMMUG00000014658	0.40	0.0992
VPS13C	Vacuolar Protein Sorting 13 Homolog C	ENSMMUG00000001362	0.33	0.0662
CDC42BPA	CDC42 Binding Protein Kinase Alpha	ENSMMUG00000008638	0.32	0.0942
ERC1	ELKS/RAB6-Interacting protein	ENSMMUG00000010933	0.31	0.0992
**Downregulated genes**				
DDT	D-Dopachrome Tautomerase	ENSMMUG00000004552	−1.07	0.0891
PHB2	Prohibitin	ENSMMUG00000010205	−0.58	0.0548
CCT8	Chaperonin Containing TCP1 Subunit	ENSMMUG00000003023	−0.37	0.0931

Genes surpassing a significance threshold of an adjusted p-value < 0.1 in the interaction and also showing the same direction of log fold change in expression in the individual treatment contrasts “*T* vs *C*” and “*T* + *WSD* vs *WSD*” were considered significant synergistic genes. Log2Ratio” is calculated as Log2 (fold change) in gene expression.

### Correlation between gene expression and visceral adipocyte size

**Figure 2:**
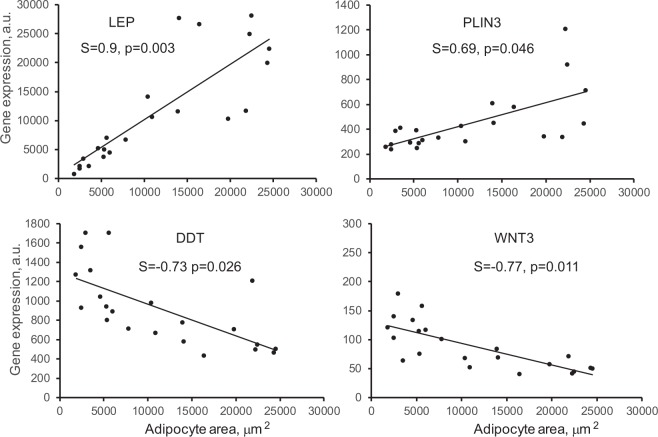
Correlation between omental adipocyte area and gene expression. Gene expression is indicated in arbitrary units (A.U.). Linear regression was determined for the combined pool of 24 samples (4 groups). Spearman correlation coefficient (S) and adjusted p-values are indicated.

We previously demonstrated that *T* + *WSD* treatment leads to an increase in visceral adiposity and omental adipocyte size^35,36^. We therefore tested if the differential gene expression described in the present study is related to adipocyte size. Using a Spearman correlation analysis, we found that the expression of 235 genes exhibited significant association with adipocyte area (adjusted p < 0.05; Supplementary Table S7). Consistent with a previous report^49^, the expression of the leptin (*LEP*) gene correlated positively with omental adipocyte area (Fig. 2). Similarly, the expression of *PLIN3* showed a positive association with adipocyte area. In contrast, the expression of *DDT* and *WNT3* genes correlated negatively with adipocyte area (Fig. 2). Interestingly, *DDT* expression exhibited synergistic regulation by *T* and *WSD* (Table 1). Thus, it appears that the expression of a particular subset of genes is significantly associated with visceral adipocyte size.

### Exposure to hyperandrogenemia and WSD is associated with global changes in DNA methylation

**Figure 3:**
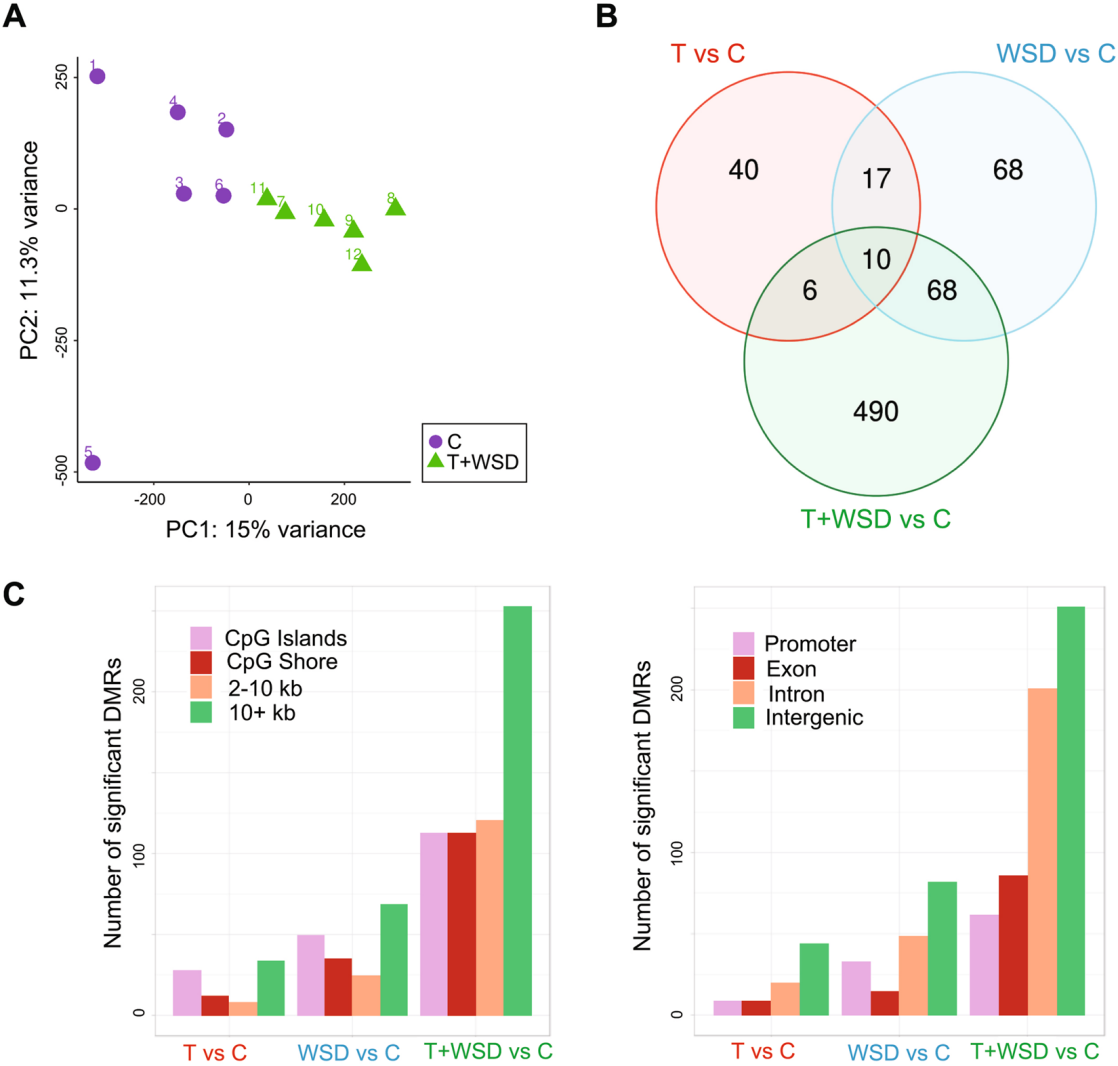
Individual and combined effects of *T* and *WSD* on DNA methylation in omental WAT. (**A**) PCA of DNA methylation for *C* and *T* + *WSD* showing the segregation of these two groups. (**B**) Venn diagram representing the overlap between DMRs (10% methylation difference cutoff and an adjusted p-value cutoff of 0.05) using the same independent comparisons and color coding as for the RNAseq [*T* (red), *WSD* (blue) and *T* + *WSD* (green)]. (**C**) Number of significant DMRs in various genomic features (left) and genomic regions (right).

We also examined whether DNA methylation was altered in visceral WAT as a consequence of the various treatments by performing reduced-representation bisulfite sequencing (RRBS), which allows for quantitative, single-base resolution analysis of the portion of the genome enriched in genes and CpG islands^50^. First, we observed global differences between the *T* + *WSD* and *C* groups as shown by PCA (Fig. 3A). As expected, the combination of hyperandrogenemia and WSD caused more global changes in DNA methylation than either treatment alone (Supplementary Fig. S1B). Indeed, when we identified differentially methylated regions (DMRs) between each treatment group and controls, the highest number of DMRs was found in the *T* + *WSD* group (n = 574), followed by the *WSD* (n = 163) and *T* (n = 73) groups (Fig. 3B**;** Supplementary Tables S8–S10). In the *T* + *WSD* group, 57% of the DMRs were found to overlap with genes, with a portion of them (17%) located in promoters (Fig. 3C). Pathway analysis using GOrilla^51^ indicated that genes overlapping with DMRs were enriched for cellular metabolic and cAMP-signaling GO biological processes (Supplementary Table S11), indicating an overlap with pathways enriched in DEGs (Fig. 1C). There was no significant correlation between promoter methylation and adipocyte area (Spearman correlation, adjusted p < 0.05).

### Limited correlation between differential gene expression and DNA methylation

Increased gene expression has been traditionally associated with promoter hypomethylation and gene body hypermethylation^52,53^. Since we observed both global differential expression and methylation, we sought to explore the relationship between these effects. We first investigated the global relationship between gene expression and promoter methylation in our study groups. To do this, we considered genes from the differential gene-expression analysis that also possessed at least one CpG with a minimum of 10X coverage in the promoter region (i.e., 3 kbp upstream from the transcription start site (TSS)). This resulted in about 11,000 genes for which we could define both an expression value and an average promoter methylation percent value. We observed a trend for an inverse correlation between gene expression and promoter methylation within each sample group (Spearman correlation value of approximately −0.2) (Supplementary Fig. S3).

We then identified genes that were both differentially expressed and differentially methylated in response to the different treatments. Specifically, we identified 63 DEGs in the *T* + *WSD* group that also overlapped or were within 5 kb of a DMR. Of these DEGs, 12 showed differential methylation at the promoter region and 9/12 displayed an inversed correlation between methylation and expression (i.e., hypomethylation with increased expression and hypermethylation with decreased expression, Table 2). Hypermethylation in gene bodies has traditionally been associated with activation of transcription. We observed that 26 of the 54 DMRs found in gene bodies were hypermethylated and 13 of those were indeed associated with increased gene expression, while 28 genes showed a “promoter-like” inverse correlation between methylation and expression (i.e., hypomethylated DMRs and increased expression, or vice versa, Table 2). The latter relationship has been reported to correspond to alternative promoters or active enhancer regions^53^. Using the LiftOver tool from the UCSC browser, we converted these 54 DMRs from the rhesus to the human genome (hg38), for which functional annotations are available through the ENCODE project^54^. We observed that DMRs in up-regulated genes corresponded more often than expected by chance to DNase I hypersensitive sites in the human genome (p-value 0.0115, Fisher’s test, two tails), suggesting that these regions might behave as transcription factor (TF) binding sites.

**Table 2:** Correlations between gene expression and DNA methylation in omental WAT.

*T* + *WSD* vs *C*					
Symbol	Region	Methyl1	Methyl2	Log2FC	Correlation
C21orf58	P, I	−23.4		2.5	Inverse
MATN3	P	−18.5		1.2	Inverse
CD93	P, E	−18.6	−17.1	1.0	Inverse
CTXN1	P, E	20.6		−1.5	Inverse
SMTNL2	I, P	19.6	32.1	−2.0	Inverse
RNF227	P	26.9		−2.2	Inverse
TRIM29	P	19.4		−2.6	Inverse
MISP	P	20.3		−3.0	Inverse
MAB21L2	P, E	21.1		−6.5	Inverse
GATA5	P	−12.8		−1.3	Direct
SHF	P	−12.8		−1.7	Direct
SLC6A3	P	−14.4		−2.0	Direct
GUCY2D	E, I	−17.7		1.8	Inverse
CCDC3	I	−12.9		1.8	Inverse
MYH7B	I	−19.4		1.5	Inverse
ADCYAP1R1	I	−16.5		1.4	Inverse
GALNT17	I	−31.4		1.3	Inverse
EPAS1	I	−18.6	−16.1	1.0	Inverse
PMEPA1	I	−20.3		1.0	Inverse
APLNR	E	−17.1		1.0	Inverse
KIF21B	I	−14.2		1.0	Inverse
ADGRG1	I	−16.1		1.0	Inverse
PECAM1	I	−22.2		0.9	Inverse
NDST1	I	−26.4		0.9	Inverse
HSPA12A	I	−19		0.9	Inverse
MYO9B	I	−13.2		0.7	Inverse
PTPRG	I	−21.3		0.6	Inverse
RASA3	I	−19.2		0.6	Inverse
TSPAN14	I	−23		0.6	Inverse
EFNA5	I	24.3	24.4	−0.7	Inverse
BAIAP2	I	17.9	18.7	−0.7	Inverse
MST1R	E, I	18.3		−0.7	Inverse
MCU	I	34.3		−0.9	Inverse
SLC9A3R1	I	25.2		−1.2	Inverse
GATA6	E, I	23.9		−1.3	Inverse
SSTR3	I	20.7		−1.4	Inverse
C3	I	26.4		−1.6	Inverse
PDZK1IP1	E, I	26		−2	Inverse
LMO7	I	29.7		−2.5	Inverse
KRT8	I	14.5		−2.8	Inverse
IL4I1	I	24.9		2.3	Direct
ITGAX	E, I	16.2		1.8	Direct
NAV1	E, I	18.5	11.9	1.1	Direct
NEK6	I	10.3		1.0	Direct
STAB1	I	14.2		1.0	Direct
RPH3AL	I	15.5	24.4	1.0	Direct
NOS3	E	19.1		0.9	Direct
MAP7D1	E, I	13.5		0.7	Direct
GSE1	I	22		0.7	Direct
CAMKK2	I	18.7		0.7	Direct
HTRA1	E, I	19		0.6	Direct
FAM178B	I	−17.7		−1.1	Direct
ROR2	I	−18		−1.2	Direct
MKX	E, I	−13.1		−1.3	Direct
EBF4	E, I	−11		−1.4	Direct
GRM7	I	−16.7		−1.5	Direct
VIPR2	I	−15.1		−1.5	Direct
TNK1	E, I	−23.6		−2.0	Direct
WNT7B	I	−13.3		−2	Direct
S1PR5	E	−21.3		−2.3	Direct
GATA4	I	−12.5		−2.7	Direct
***WSD*** **vs** ***C***					
HOXA10	I	19		−4.0	Inverse
CCDC3	I	−14.5		1.4	Inverse

Genes with inverse or direct correlation between expression levels and DNA methylation changes in indicated genome regions. P, promoter; E, exon; I, intron; Methyl, differentially methylated region (DMR); Log2Ratio” is calculated as Log2 (fold change) in gene expression in “*T* + *WSD*” or “*WSD*” groups compared to the control (“*C*”). The DMRs are more than 1 bp in length and it is possible for them to overlap more than 1 gene region. For example, the DMR might span an exon/intron junction, or a promoter/1st exon junction.

While no association between differential methylation and gene expression was apparent for DEGs and DMRs from the *T* group, we identified two genes (*HOXA1* and *CCDC3*) that showed an inverse correlation between expression and methylation in the *WSD* group, with both DMRs being intronic (Table 2). In conclusion, although we see a large global effect of *T* + *WSD* treatment on both DNA methylation and gene expression, only a portion of differentially expressed genes also exhibited differential DNA methylation.

### *In silico* analysis of interactions between transcription factors and DMRs

**Figure 4:**
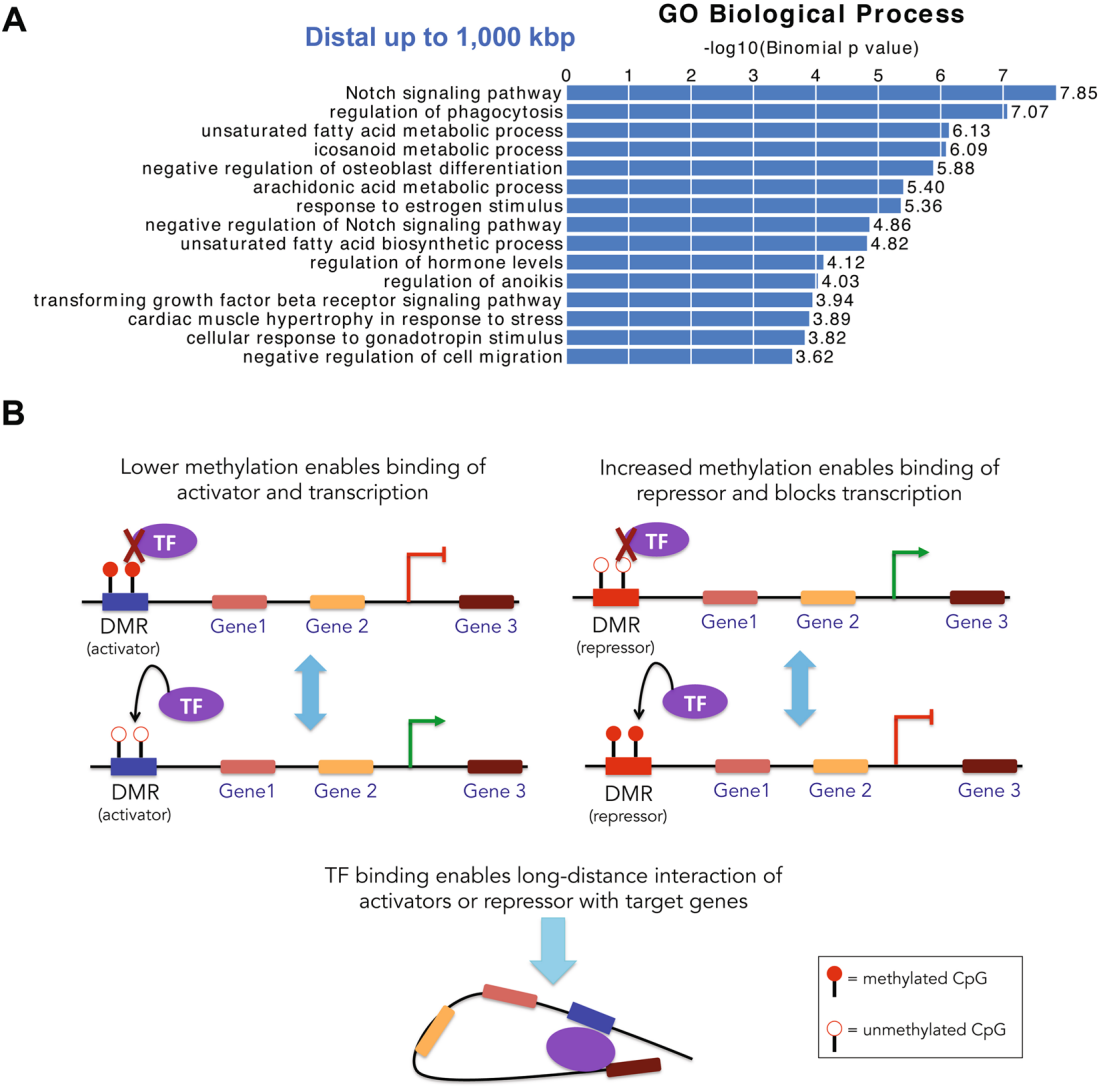
DMRs distally located with respect to genes are enriched in biologically relevant pathways. (**A**) Pathways analysis using the Genomic Regions Enrichment of Annotations Tool (GREAT) that associates each gene with a ‘regulatory domain’ defined as a genomic region 5 kb upstream and 1 kb downstream from the TSS and an extension within 1 Mb up to the regulatory domain of the nearest upstream or downstream gene. (**B**) Schematic model depicting methylation-dependent modulation of TF binding to distal regulatory domains. Left diagram, hypermethylation of an *activator* inhibits, while hypomethylation of an *activator* facilitates TF binding, resulting in transcriptional repression or activation, respectively. Right diagram, particular TFs bind hypermethylated repressors, leading to transcriptional repression, while hypomethylation has the opposite effect on transcription. Bottom diagram, TF binding to a regulatory domain enables long-distance interactions with the target genes through 3D chromatin structural rearrangement.

Studies show that interactions between TFs and promoter regions can occur over long genomic distances through 3D chromatin structural rearrangement^55^. Given the lack of a strong correlation between differential methylation and differential gene expression reported in the present study, we hypothesized that a portion of DMRs can bind TFs and act as distal regulators of gene expression. To test this hypothesis, we employed *in silico* analysis using the Genomic Regions Enrichment of Annotations Tool (GREAT) that associates each gene with a ‘regulatory domain’ defined as 5 kb upstream and 1 kb downstream from the TSS and an extension within 1 Mb up to the regulatory domain of the nearest upstream or downstream genes^56^. While no pathways were enriched in the *T* and *WSD* groups, the analysis identified several pathways of interest in the *T* + *WSD* group, including those regulating notch signaling, unsaturated fatty acid metabolism, and estrogen response (Fig. 4A). However, when we excluded long-distance regulatory domains (i.e., 1 Mbp) from the analysis, these pathways were no longer detected, suggesting that these biologically relevant processes are enriched for DMRs that act as distal regulators (Supplementary Fig. S4).

To identify potential TF binding motifs involved in distal regulation, we performed *in silico* motif enrichment analysis using the Hypergeometric Optimization of Motif Enrichment (HOMER) bioinformatic tool^57^. We identified several significantly enriched motifs in the DMRs from the *T* + *WSD* group (Supplementary Table S12), while no significant enrichment was found for the *T* and *WSD* groups. Among the significantly enriched motifs identified in the present study, one interesting example is the Recombination Signal Binding Protein for Immunoglobulin Kappa J (RBPJ, Supplementary Table S12). Using BLASTn, we determined that the nucleotide sequence of the rhesus macaque *RBPJ* gene is 95% identical to human. Furthermore, BLASTp analysis showed that human and rhesus RBPJ proteins are 99% identical. This provides strong support that our RBPJ motif enrichment translates to our rhesus model. Using EnrichR^58^, we also determined that the promoters of DEGs in the *T* + *WSD* group were significantly enriched for the RBPJ binding motif, suggesting that the differential binding of this TF may regulate gene expression. Thus, this computational analysis suggests that DMRs may act as distal regulators through binding of biologically relevant TFs and that changes in methylation due to treatment (*T* + *WSD*) may modulate the binding of TFs, ultimately impacting gene expression (Fig. 4B).

## Discussion

One of the many challenges that hinders our understanding of the pathophysiology of PCOS is dissecting out the relative contribution of hyperandrogenemia and diet in the etiology of this multifactorial disease. The role of hyperandrogenemia and diet-induced obesity as the potential co-drivers of metabolic, adipose-specific^35,36^, and reproductive^38,39^ phenotypes has been demonstrated in our recent NHP studies. These studies established that the combination of hyperandrogenemia and WSD induces greater metabolic (obesity and insulin resistance) and adipose-specific (visceral adipocyte hypertrophy, increased lipid storage, and reduced lipolysis) dysfunction than either treatment alone^35,36^. These and other findings^32–34^ raise the intriguing possibility that hyperandrogenemia alone does not induce weight gain and metabolic dysfunction in young females unless combined with an obesogenic diet.

Our previous NHP studies demonstrated that the combination of hyperandrogenemia and WSD induces intra-abdominal visceral adiposity, as evidenced by the significant enlargement of omental adipocytes^36^. An increase in visceral adiposity has previously been linked to the pathophysiology of insulin resistance in PCOS patients^10–12^, while the mechanisms remain poorly understood. One possibility is that the combination of hyperandrogenemia and WSD induces the masculinization of fat depots in females, leading to increased lipid storage in visceral WAT and decreased lipid storage in subcutaneous WAT^59^. The latter plays a positive role in metabolism, while the redirection of lipid stores from the subcutaneous to the visceral WAT depot is associated with metabolic syndrome^60^. Mechanistically, hyperandrogenemia in the presence of WSD increases insulin-stimulated FFA uptake and inhibits lipolysis in omental WAT, favoring the development of intra-abdominal obesity in NHPs^36,59,61^, while the mechanism remains poorly understood.

The present study was designed to address the potential mechanisms regulating visceral adiposity in the NHP model. We first showed that *T* + *WSD* induces a greater transcriptional response in omental WAT than either treatment alone. Interestingly, several of the DEGs that we detected in rhesus visceral WAT, including *DKK2, SVEP1, NRCAM, GPT*, and *DMAP1*, were previously reported to be differentially expressed in subcutaneous WAT of PCOS women^42^, confirming that our model is recapitulating some of the transcriptional changes observed in this disease, albeit in different depots. In addition, we observed that *T* + *WSD* treatment induced the upregulation of several lipid metabolism genes, including the fatty acid transporter *CD36*, fatty acid binding protein 4 (*FABP4*), triglyceride synthesis enzyme *MOGAT1*, and lipid droplet protein perilipin 3 (*PLIN3*), which collectively have a positive effect on FFA uptake and triglyceride storage in WAT. Importantly, the expression of the *MOGAT1* and *CD36* genes in omental WAT and circulating levels of FATP4 have been shown to be elevated in PCOS women^42,62,63^. In agreement with our RNAseq results, the expression of the fatty acid transporter *CD36* has been shown to correlate with omental adipocyte size in women with PCOS^63^. Furthermore, our previous studies have demonstrated that *T* + *WSD* treatment enhances FFA uptake in omental WAT of NHPs^36,61^. We also observed that genes encoding the components of the LXR/RXR pathway were upregulated in *T* + *WSD*, suggesting potential changes in lipoprotein metabolism. This is consistent with a study of WAT in women with PCOS^40^. The increased expression of genes that regulate HDL metabolism and reverse cholesterol transport (e.g., *ABCA1*, *ABCG1*, *ApoA1*, *APOD*, *CETP*, *PLTP*, *PON3*, and *SR-A*) is compatible with other well-known effects of LXR activation, including cellular cholesterol accumulation^64–66^.

Our data indicate that hyperandrogenemia may attenuate cAMP-dependent lipid catabolism. The breakdown of cellular triglycerides depends on the activation of the cAMP-dependent hormone sensitive lipase (HSL), the principal lipase responsible for the β-adrenergic lipolytic response in WAT^67^. The HSL gene (*LIPIE*) has been shown to be significantly downregulated in omental WAT from *T* + *WSD*-treated NHPs^61^ and PCOS women^63^. In line with these observations, we showed that hyperandrogenemia induces lipolytic resistance in omental and subcutaneous WAT of NHPs^36,61^. In contrast, omental WAT from PCOS women exhibits increased catecholamine-induced lipolysis^68^, while subcutaneous WAT exhibits decreased catecholamine-induced lipolysis and the reduced expression of the β_2_-adrenergic receptor^18^, the regulatory II β-subunit of protein kinase A, and HSL^19^. Although the present study did not detect differential expression of the *LIPE*, *PRKCA* (catalytic subunit of protein kinase A), and *ADRB2* (*β*_*2*_*-*adrenergic receptor) genes, several isoforms of phosphodiesterase (PDE) that mediate cAMP degradation were significantly upregulated in the *T* + *WSD* group. This finding agrees with an earlier report showing the upregulation of several PDE genes in omental WAT of PCOS women^49^. Furthermore, clinical studies demonstrated that the PDE4 inhibitor roflumilast added to metformin reduced fat mass in obese women with PCOS^69^, suggesting that hyperandrogenemia may lead to decreased cAMP levels in WAT.

There is growing evidence that body fat distribution is dynamically regulated at the transcriptional and DNA methylation levels^44–46,70–74^. For example, obesity induces DNA hypermethylation in the promoter of the adiponectin gene, resulting in its reduced expression and the development of insulin resistance in mice^72^. Similarly, DNA methylation of the perilipin-1 promoter is higher and gene expression is lower in WAT of obese subjects^70^. Thus, we sought to test whether hyperandrogenemia and WSD regulate the transcriptional response in WAT through the DNA methylation mechanisms. Using a genome-wide approach, we show that all treatments elicit global changes in DNA methylation, with *T* + *WSD* causing the largest effect. Although we observed a trend of global inverse correlation between methylation and transcription, only 63 genes exhibited both significant differential expression and DNA methylation. Of note, in a genome-wide study of gene expression and DNA methylation in subcutaneous WAT of PCOS women, 33 DEGs that also displayed differential DNA methylation were identified^42^, although none of these 33 genes were found to overlap DMRs in our study. A limited association between gene expression and DNA methylation is not entirely surprising, as the relationship between DNA methylation and gene expression is more complex than originally thought^53^. For instance, the functional role of DNA methylation in gene bodies is still elusive^53^. It is also possible that DNA methylation is regulated by WAT depot-specific mechanisms. In the original study, Xu and colleagues reported that prenatally androgenized rhesus macaques display altered genome-wide DNA methylation in visceral WAT^75^. In spite of significant differences in methodology and dietary regiments used by their and our groups, both studies identified 3 common DMRs associated with the *CCDC3, GRM7*, and *NEK6* genes. Importantly, the present study showed that these genes are also differentially expressed in rhesus WAT. *CCDC*3 has been previously shown to be upregulated in omental WAT of obese subjects and exerts adipogenic effects *in vitro* and *in vivo*^76,77^. In the present study, *CCDC3* was found to be upregulated and differentially methylated at the same intronic region by either *WSD* or *T* + *WSD* treatment, suggesting a diet-specific mechanism of gene regulation. Based on our GREAT pathway analysis, we hypothesized that a portion of the DMRs may be acting as distal regulators, rather than acting on the closest gene. Indeed, we observed that DMRs are not enriched in biologically relevant pathways, like the Notch signaling pathway, when long-distance relationships (1 Mb) were excluded in looking at *cis*-interactions between DMRs and genes. Moreover, our computational analysis showed that DMRs from the *T* + *WSD* group were enriched in TF binding motifs, supporting the regulatory role of DNA methylation in modulating TF binding affinities^78^. As an example, RBPJ has been shown to control the expression of notch signaling genes^79^ and to bind chromatin in a methylation-dependent fashion^80,81^.

Our study does have limitations. First, we did not evaluate transcriptional and epigenetic responses in isolated adipocyte vs stromal-vascular cell populations due to insufficient sample size for separation of individual cell types. However, previous analyses of human WAT also employed total tissue samples; thus, comparison of our NHP-derived data from whole WAT to these data is appropriate. Second, we did not examine transcriptional and epigenetic changes in subcutaneous WAT because this depot is insufficiently developed in young macaques to obtain enough tissue. Third, the number of animals in each group (n = 6) was relatively small. However, given a unique nature of this NHP model, the present study provides valuable translational information on the interactions between obesity and hyperandrogenemia in PCOS. Fourth, other epigenetic modifications (e.g., histone modifications and micro-RNAs^82,83^) may also contribute to WSD and T effects and need to be studied in order to fully understand the mechanisms underlying transcriptional changes observed in the present report.

In conclusion, this study demonstrates that the combination of hyperandrogenemia and WSD induces a synergistic transcriptional response associated with an increase in lipid anabolic capacity in the visceral WAT of NHPs^36,61^, similar to findings reported in women with PCOS^10–12^. This and other PCOS-related studies^40,42^ demonstrate a limited association between WAT gene expression and DNA methylation. Furthermore, the majority of genes showed no significant association with adipocyte size based on mRNA or promoter methylation levels, suggesting that hyperandrogenemia but not adipocyte hypertrophy per se is the primary etiological factor driving the transcriptional and epigenetic response in WAT. Thus, the intake of excess dietary lipids in combination with hyperandrogenemia may facilitate lipid accumulation and the development of visceral adiposity. Collectively, the results of this study support the need for future clinical trials aimed at early dietary interventions to prevent weight gain in young women with PCOS, and in developing strategies aimed at maintaining weight loss in obese women with PCOS.

## Methods

### Animal model

All animal procedures were approved by the Oregon National Primate Research Center (ONPRC) Institutional Animal Care and Use Committee and comply with the Animal Welfare Act and the APA Guidelines for Ethical Conduct in the Care and Use of Nonhuman Animals in Research. Animal characteristics and the origin of WAT samples used in the present study have been previously described^35,36^. Briefly, female rhesus macaques were selected for the study and randomly assigned to one of four treatment groups (*C, T, WSD* and *T* + *WSD*). T-releasing capsules were prepared and implanted subcutaneously as previously described^35^. Animals were maintained *ad libitum* on either chow diet consisting of two daily meals of Fiber-balanced Monkey Diet (15% calories from fat, 27% from protein, and 59% from carbohydrates; no. 5052; Lab Diet, St. Louis, MO), supplemented with fruits and vegetables, or a WSD, containing 36% calories from fat, 18% from protein, 45% from carbohydrates (TAD Primate Diet 5LOP, 5A1F, Lab Diet). Animals had undergone 3 years of continuous treatment and were approximately 5.5 years of age and were post-pubertal at the time of WAT biopsy collected as previously described^36^. To perform gene expression and DNA methylation analyses, we selected 24 animals (6 representative animals from each group [*C, T, WSD*, and *T* + *WSD*]) that reflected the overall group findings^36^; i.e., increased fat mass and hyperinsulinemia at 3 years of treatment. Supplementary Table S1 provides these metabolic characteristics previously published, but for the specific subset of animals (n = 6/group) utilized in the current study.

### RNA-seq libraries

Adipose biopsy procedures were previously described^36^. DNA and RNA were extracted from 200 mg of frozen visceral (omental) WAT homogenized using a TissueLyzer-II (QIAGEN, Hilden, Germany) with the AllPrep DNA/RNA purification kit (QIAGEN). High-quality RNA samples (RIN > 8) were used for library construction using the TruSeq Rybo-zero method and were sequenced by the Massively Parallel Sequencing Shared Resource (MPSSR) at OHSU using the Illumina HiSeq. 2500 platform, with the 100-bp, single-read protocol. RNA-seq summary statistics is shown in Supplementary Table S13.

### Differential expression and synergy analysis

Sequencing reads were evaluated with FastQC (v0.11.5)^84^ and trimmed to remove adapters and low-quality regions using Trimmomatic (v0.36)^85^. After trimming, reads were aligned on the most current rhesus macaque genome assembly (rheMac8) using STAR^86^ with default parameters. DESeq. 2 (v1.18.1)^48^ was used for differential expression analysis of the count data. Pairwise comparisons were performed among the sample groups and Benjamini-Hochberg adjustment was used to adjust the p-values for multiple comparisons when performing statistical tests on thousands of genes. The lists of differentially expressed genes (FDR < 0.05; −1.5 > Fold Change > 1.5) are reported in Supplementary Tables S2–S4. Differentially expressed genes were analyzed with Ingenuity Pathway Analysis (IPA) to identify pathways enrichment. In order to find genes for which the combined *T* + *WSD* treatment might have had a synergistic effect on gene expression, we fit a 2 × 2 factorial design and looked for genes showing significance in the interaction term of *T* and *WSD* utilizing the DESeq. 2 package. This allowed us to determine genes that had a change in expression due to T treatment that was also dependent on diet condition. Genes surpassing a significance threshold of adjusted p-value < 0.1 in the interaction and also showing the same direction of log fold change in expression in the individual treatment contrasts “*T* vs *C*” and “*T* + *WSD* vs *WSD*” were considered significant synergistic genes. We additionally identified genes possibly showing a synergistic effect due to treatment for which the unadjusted p-value was less than 0.1 (Supplementary Table S6). To test the association between gene expression and adipocyte area, we applied a Spearman correlation analysis using ~19,000 genes which passed low count filtering to be included in our differential expression analysis pipeline.

### Reduced-representation bisulfite sequencing (RRBS)

RRBS libraries were generated from ~200 ng of WAT genomic DNA following an established protocol^50^. Briefly, overnight digestion was performed with *MspI* (New England Biolabs, Ipswich, MA), which cuts the sequence CCGG and generates sticky ends, enabling every read to start with a CpG. Libraries were prepared with the NEXTflex Bisulfite-Seq Kit (Bioo Scientific Corporation, Austin, TX) and the NEBNext Methylated Adaptors (New England Biolabs). The ligated DNA was size-selected using AMPure XP magnetic beads to produce a final library size of ~350 bp. Bisulfite conversion was performed with the EZ DNA Methylation-Gold Kit (Zymo Research, Irvine, CA) before carrying out PCR amplification with NEBNext Multiplex Oligos (New England Biolabs) to barcode each library. The resulting libraries were normalized and multiplexed for sequencing on the Illumina NextSeq. 500 with the high-output, 75-bp cycle protocol. Sequencing reads were evaluated with FastQC^84^ and trimmed to remove adapters and low-quality regions with Trim Galore (v0.4.2)^87^ using the “RRBS” parameter. Trimmed reads were aligned to rheMac8 with Bismark (v0.16.1)^88^, the most widely used software for mapping bisulfite converted sequences. Summary statistics for the RRBS datasets are shown in Supplementary Table S14.

### Differential methylation analysis

In order to identify differentially methylated cytosines (DMCs) in a CpG context and differentially methylated regions (DMRs) we used a multi-step approach. First, Limma (v3.34.9)^89^ was used to perform DMC analysis, as Limma allows modeling of more complex designs. For DMC analysis, we included CpGs with at least 10X coverage in at least 4 of the 6 replicates per group, resulting in 841,143 CpG sites. For input values, we performed an arcsine transformation on the methylation rate per CpG. Then, we used the p-values obtained from the DMC analysis as input to Comb-p^90^ in order to find DMRs. DMR regions are found by seeding on CpGs with corrected p-values < 0.05 and extending the region as long as it finds another CpG with a corrected p-value < 0.05 within 300 bp. All DMRs (Sidak p-value < 0.1; methylation difference > 10%) and their annotations are listed in Supplementary Tables 8–10. As many DMRs might not overlap with genes or their promoters (we annotated them as “inter-genic”) but might correspond to distal regulatory elements, we also annotated the closest transcription start site (TSS) for each inter-genic DMR. In order to identify pathways enriched in DMRs, we used the publicly available tools GREAT (http://great.stanford.edu/public/html/)^56^ and GOrilla (http://cbl-gorilla.cs.technion.ac.il/)^51^. Finally, motif enrichment was performed on the significant DMRs from the three main comparisons using HOMER (Hypergeometric Optimization of Motif EnRichment)^57^, specifying the use of the given size of the regions and normalizing for CpG content against the random background. Although the motif databases used by HOMER might be skewed towards human and mouse, TF binding motifs are highly conserved and therefore interchangeable between mammals and vertebrates in general^57^.

## Supplementary information

Supplementary Information

Supplementary Tables S1-S14

## Data Availability

Gene expression and DNA methylation data are available at the NCBI Gene Expression Omnibus data repository under Accession Number GSE124709.
