# Supplementary material for: Synergistic Effects of Hyperandrogenemia and Obesogenic Western-style Diet on Transcription and DNA Methylation in Visceral Adipose Tissue of Nonhuman Primates
Source: Sci Rep. 2019 Dec 17;9:19232. doi: 10.1038/s41598-019-55291-8 (PMC6917716; doi:10.1038/s41598-019-55291-8)
Supplement: Supplementary file 1 — Supplementary Information [file 41598_2019_55291_MOESM1_ESM.pdf]

Supplementary Information

Synergistic Effects of Hyperandrogenemia and Obesogenic Western-style Diet on Transcription and DNA Methylation in Visceral Adipose Tissue of Nonhuman Primates

Lucia Carbone[1,2,3,4#], Brett A. Davis[2#], Suzanne S. Fei[4], Ashley White[5], Kimberly A. Nevonen[1], Diana Takahashi[5], Amanda Vinson[1,3,4,5], Cadence True[5], Charles T. Roberts, Jr.[5,6], Oleg Varlamov[5*]

# these authors contributed equally to the paper

Departments of [1]Medicine, [1]Knight Cardiovascular Institute, [2]Molecular and Medical Genetics, and [3]Medical Informatics and Clinical Epidemiology, Oregon Health & Science University, Portland, OR, USA; Divisions of [4]Genetics, [5]Cardiometabolic Health, and [6]Reproductive and Developmental Sciences, Oregon National Primate Research Center, Beaverton, OR, USA

*Corresponding author: Oleg Varlamov, varlamov@ohsu.edu

Supplementary Fig. S1 PCA plots including all four groups for A) RNA-seq data and B) RRBS data. These plot show separation of the groups and complement the plots shown in Figures 1 and 3, in which only *C* and *T+WSD* were shown.

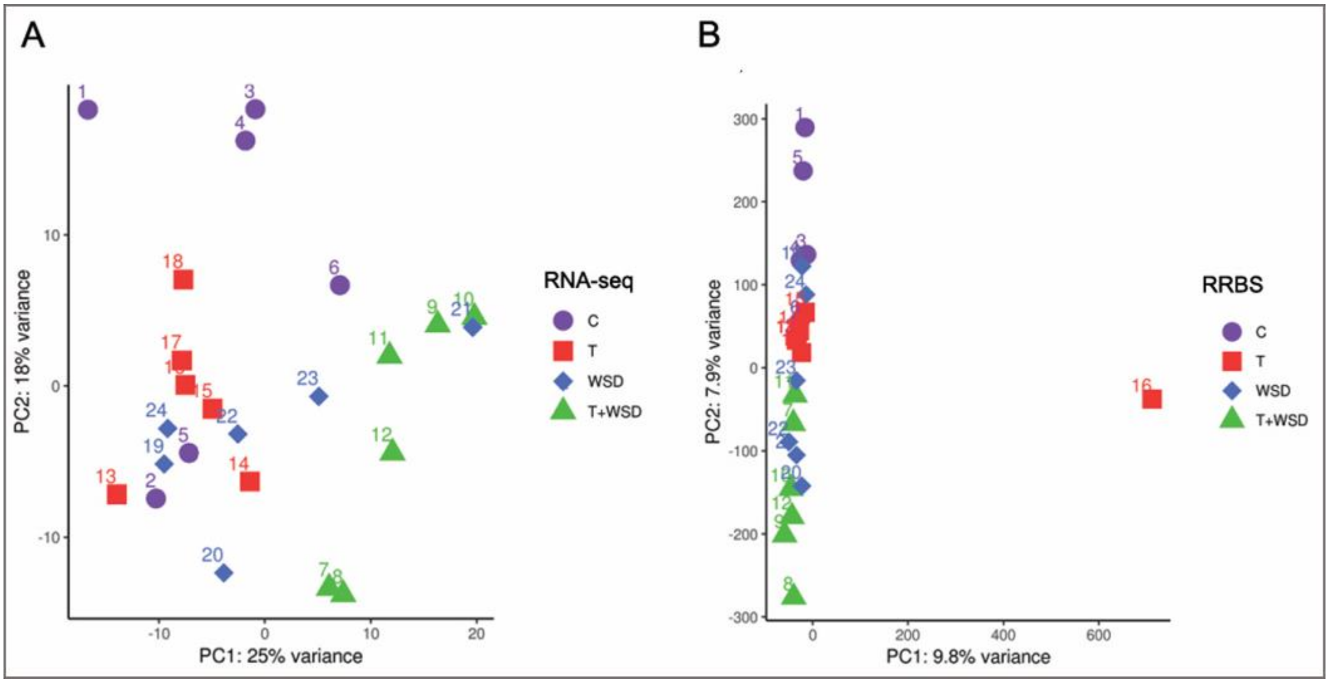

Supplementary Fig. S2 Synergistic effect of WSD and T on gene expression. A) The plots show the different effects of *T*, depending on diet for a subset of genes showing a significant synergistic effect of the combined treatment of *T* and *WSD*. The x-axis represents the absence or presence of T treatment, while the y-axis represents DESeq2 normalized count values. Each small point on the plot represents a sample, and the mean count value among treatment group replicates is represented with a larger point. As shown on the upper right, the different slopes of the two lines illustrate the interaction between *T* and *WSD*: in the absence of interaction, the two lines will have roughly equal slopes. B) Pathways identified using EnrichR enriched in genes showing a trend for being synergistically down-regulated (red arrow) or up-regulated (green arrow).

A

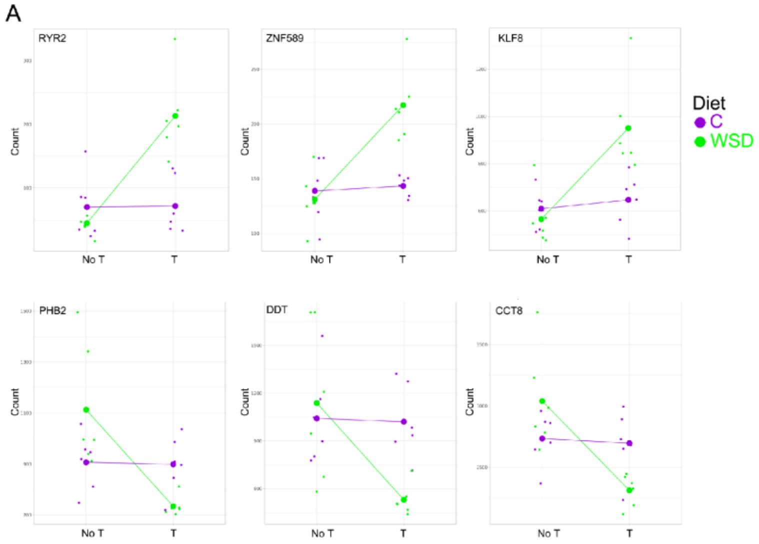

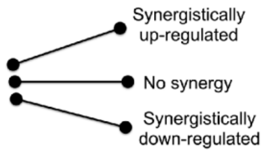

B

Pathway	Adj p-value	Combined score	Direction
Cholesterol Biosynthesis Pathway	0.0004508	37.18	⬇
Proteasome Degradation	0.00000141	35.38	⬇
Glycolysis and Gluconeogenesis	0.0002661	23.55	⬇
Mitochondrial complex I assembly model OXPHOS system	0.0005648	20.75	⬇
Sterol Regulatory Element-Binding Proteins (SREBP) signaling	0.0002661	19.35	⬇
Serotonin Receptor 2 and ELK-SRF/GATA4 signaling	0.03167	23.22	⬆
Hedgehog Signaling Pathway	0.03167	21.28	⬆

Supplementary Fig. S3 Scatterplots of gene expression vs promoter methylation for the 4 sample groups. The plots include gene expression values (log TPM averaged across replicates) and promoter methylation values (averaged across replicates) for 11,333 genes that passed low count filtering in the differential gene expression analysis and also contained at least 1 CpG with a minimum of 10X coverage in the promoter region. The spearman rank correlation indicated a slightly negative correlation between gene expression and promoter methylation, with rho values near -0.2 for each sample group.

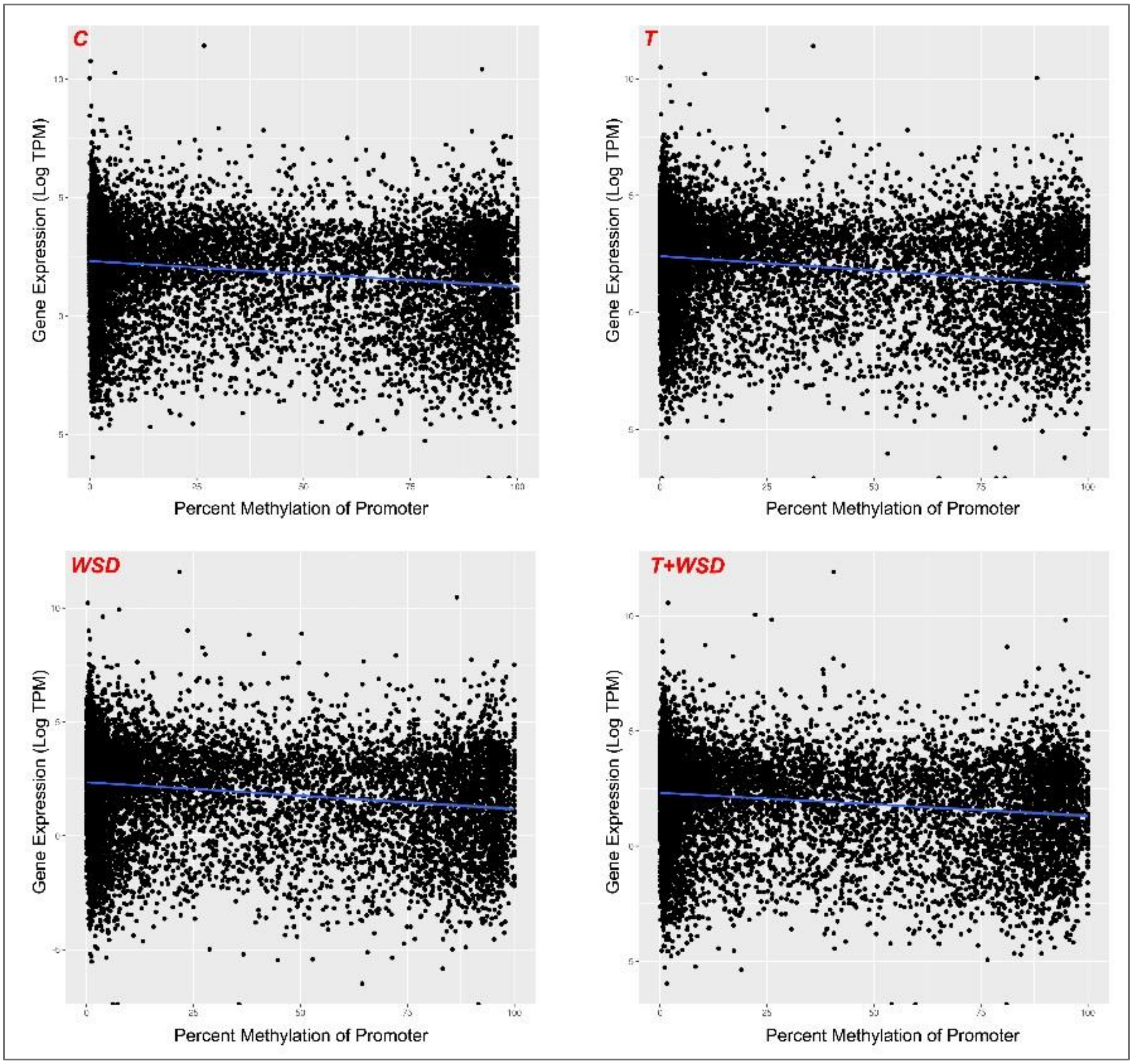

Supplementary Fig. S4 Pathways enriched in DMRs when distal (<1Mb) relationships are included (left) are biologically relevant to the observed phenotype and differentially expressed genes. When distal relationships are excluded (right) more generic pathways are found to be enriched, indicating that regulation of specific genes occurs through long distance relationships between DMRs and genes.

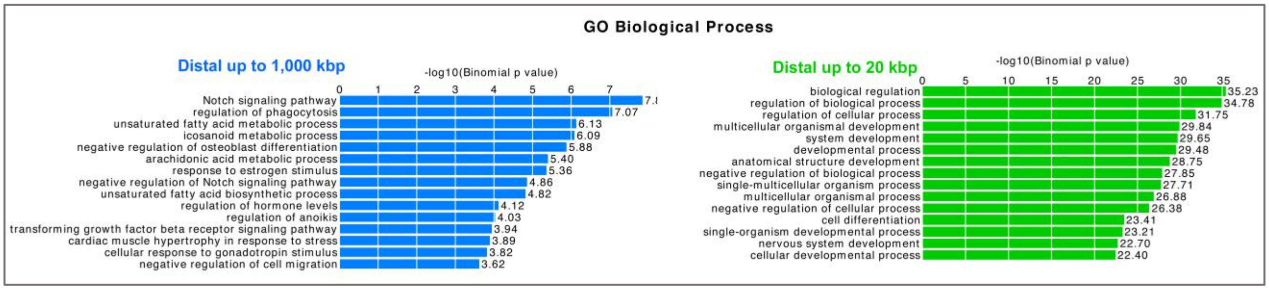

Description of Supplementary Tables S8-S10

Supplementary tables S7-S9 contain the significant DMRs (Sidak p-value < 0.1 and absolute methylation difference > 10%) for each of the 3 comparisons. Each DMR has a “DMR.ID” consisting of its “chr_start_meth.diff” information. Columns B-C provide the chromosome, start, and end position of the DMR. Columns E-H provide the length of the DMR (region_length), the number of CpGs within the DMR (num_cpgs), the percent methylation difference of the DMR in the comparison (meth.diff), and the Sidak p-value for the DMR. The methylation difference is calculated as the average percent methylation value of the region in the treatment samples minus the average percent methylation difference of the region in the control samples. We then provide annotation for the DMRs using Ensembl version 92 annotation for the Mmul8 reference genome. Columns I-K provide whether the DMR overlaps an annotated promoter (3 kb upstream from TSS), exon, or intron with a value of “1” signifying that an overlap is present. If there is no overlap for a promoter, exon, or intron, then the DMR is labelled as intergenic in column L. If the DMR overlaps an exon or intron, we provide the Ensembl Gene ID of that gene in column M; column M has a value of “NA” if the DMR does not overlap an exon or intron. If the DMR overlaps a promoter, we provide the corresponding Ensembl Gene ID in column N; column N has a value of “NA” if no promoter overlap is present. If the DMR does not overlap an exon or intron, we provide the distance to the nearest TSS in column O and also the Ensembl Gene ID of the gene with the nearest TSS in column P; columns O and P have a value of “NA” if the DMR overlaps an exon or intron. Finally, we provide 5 columns of gene information taken from Ensembl Biomart corresponding to the relevant Ensembl Gene ID (if present for the DMR). Columns Q-U correspond to the “gene overlap” Ensembl Gene ID (column M), columns V-Z correspond to the “promoter overlap” Ensembl Gene ID (column N), and columns AA-AE correspond to the “nearest TSS” Ensembl Gene ID (column P).

Supplementary Methods

IPA analysis

Differential expression results (fold change, p-value, and false-discovery rate (FDR)) were imported into the Ingenuity Pathway Analysis program (IPA, QIAGEN, Hilden, Germany). To facilitate this, Ensembl rhesus gene IDs were first mapped to human gene IDs using Ensembl's BioMart. Only high-confidence matches were used, and only the top match was chosen when one rhesus ID mapped to many human gene IDs. If no human gene ID was found, the rhesus gene ID was retained. Of the 19,063 genes quantified by the experiment, 15,676 (82%) mapped into IPA's knowledgebase. Once imported into IPA, a fold-change cutoff of 1.5 and an FDR cutoff of 0.05 was applied to all comparisons to select differentially expressed genes.
